# Ready-made bodily sensations

**DOI:** 10.1038/s41598-025-14061-5

**Published:** 2025-08-18

**Authors:** Nicole Ruta, Gemma Schino, Brendan Wolfe, Marina Iosifyan

**Affiliations:** 1 School of Divinity, University of St Andrews, St Mary’s College, St Andrews, KY16 9JU Scotland; 2 Department of Psychology, University of Groningen, Groningen, 9712 TS The Netherlands; 3 Aesthetics and Learning Lab, Leibniz-Institut für Wissensmedien (IWM), 72076 Tübingen, Germany

**Keywords:** Psychology, Human behaviour

## Abstract

Embodiment plays an important role in art engagement, yet it is unclear how expectations shape bodily sensations, especially when interacting with ready-made art. In this study, we investigated how expectations and the nature of images of everyday objects affect bodily sensations. We tested if bodily sensations changed depending on (1) whether the participants were told that everyday objects were from a Museum, Commercial or Mixed context and (2) the nature of the images, counterbalancing whether they were of ready-made art or not. To measure bodily sensations, we asked participants to self-report their feelings of activity getting stronger (activations) or weaker (deactivations) in their body by clicking on body silhouettes after viewing the images, a methodology called bodily sensation mapping (BSM). We found that bodily activity in the Chest and Upper Limbs areas was influenced by expectations induced by context only. At the same time, Head and Abdomen activations and Chest, Lower and Upper Libs deactivations were solely impacted by the image type. Our innovative Open Science approach to BSMs image analysis revealed how both context and image type were influenced by the activity type, contributing and enriching the ongoing debate regarding the uniqueness of art experiences and aesthetic cognition.

**Supplementary Information:**

The online version contains supplementary material available at 10.1038/s41598-025-14061-5.

## Introduction

Artworks that depict goal-directed actions, expressive facial emotions, and distinctive bodily postures often engage viewers by evoking embodied responses to the actions and emotions and postures represented^1,2^. As modern art evolved, artists began challenging the very notion of what a work of art is by incorporating everyday objects in their work. Ready-mades (or found art) are everyday objects—often created for a specific, functional, non-artistic purpose—that artists deliberately choose to assign to the status of artwork, usually by recontextualising these objects by placing them in a museum or exhibition space^3^. Classic and now iconic examples of ready-mades are Duchamp’s Bicycle Wheel^4^ consisting of a bicycle fork with a front wheel mounted upside-down on a wooden stool, or Picasso’s Bull’s Head^5^ consisting of a bicycle seat and handlebars arranged to resemble a bull’s head. This contemporary art practice bridges artistic engagement with practical associations by exploiting the ability of the objects to evoke a sense of instrumental action rooted in viewers’ everyday interactions with the exhibited items. Ready-mades offer a unique opportunity to study how everyday objects, when presented as art, can evoke different physical responses based on how much they remind us of their original function (or affordance) within an art setting.

Embodied cognition offers a useful theoretical framework to better understand our engagement with ready-made art as it highlights the essential role that bodily sensations play in all cognitive processes^6,7^. In philosophy, phenomenologists such as Merleau-Ponty^8^Dewey^9^ and von Hildebrand^10^ significantly contributed to theorising the essential role that the body plays in cognition^11^. According to von Hildebrand^10^ knowing an object (e.g., a painting or a sculpture) corresponds to understanding the creative process behind it. This idea later found strong support in scientific research, starting with Gibson’s ecological theory of perception^12^. Gibson conceptualised perception as an active engagement with our environment and was the first to introduce the term affordances, defined as the possible actions associated with or involving a certain object (e.g., a bottle affords drinking)^12^. More recently, Rietveld broadened the concept of affordances beyond physical interactions, to include our cognitive engagement with the world— such as the act of reflecting and imagining Rietveld^13^.

Advances in psychology and neuroscience led to the discovery of mirror neurons^14,15^ and the development of embodied simulation theory^16^. Research has shown that the mirror neuron system activates not only when we execute movements but also when we observe others performing the same movements^2,17^. Building on these discoveries, embodied simulation theory emphasized that our sensory-motor system is crucial not only for interacting with the world (e.g. moving, reaching, or feeling), but also for understanding others and for the act of imagining. Taken together, embodied and environmentally embedded approaches highlight the need to integrate the neuroscientific study of the brain with rigorous investigations of how the body acts and interacts with the world^18^.

Bodily Sensation Maps (BSMs) are a recently developed self-reporting tool for bodily topography aimed at assessing bodily feelings^19,20^. BSMs rely on interoception, defined as a person’s self-awareness and ability to report their own bodily changes, and aim to capture one’stheir embodied and somatic responses to stimuli or events. Bodily Sensation Maps (BSMs) allow individuals to indicate regions on whole-body silhouettes where they experience increased (activations) or decreased (deactivations) sensory activity. Previous studies have used BSMs to identify unique activation patterns across various contexts, populations, and stimuli^21–25^ including responses to narrative texts, images, and artworks^26,27^. BSMs have proven valuable as biomarkers for bodily sensations related to various cognitive, homeostatic, or even illness-related states^20^ confirming their usefulness and versatility as research tools. When people engage with BSMs and focus on reporting their bodily sensations, they activate what is referred to in the embodied cognition literature as body schema— the dynamic sensorimotor representation of one’s own body activated when experiencing and interacting with the environment^28^. The body schema is not only associated with how individuals perceive and represent their own body, but it can also be activated by tools and their associated actions, effectively extending bodily representation to objects encountered in the environment^29^. Crucially, this activation occurs whether the actions are physically executed or merely imagined^30,31^ in line with evidence from research on the mirror neuron system and embodied simulation theory. More broadly, a schema refers to ‘a collection of basic knowledge about a concept or entity that serves as a guide to perception, interpretation, imagination, or problem solving’^32^. This theoretical concept is particularly relevant to our research, as it provides a useful framework for understanding how embodied cognition extends to art experiences.

In the context of art experience, Freedberg and Gallese^2^ suggested that when we engage with a painting or sculpture, our motor system automatically simulates the artist’s gestures by mentally recreating the movements performed to make the brushstrokes or to carve the shape of the sculpture. A canonical example used to illustrate the role of embodiment in art perception is Lucio Fontana’s Concetto Spaziale series, consisting of monochromatic paintings that exhibit vertical cuts through the surface of the canvases. Embodied simulation theory predicts that, when looking at Fontana’s paintings, the viewers will mentally simulate the action of cutting through the canvas, mirroring the motor action the artist performed. Umiltà et al.^33^ observed mu-rhythm suppression in response to Lucio Fontana’s canvas but not to graphically modified versions of his paintings. The mu-rhythm suppression is an index of mirror neuron activity—often observed via an electroencephalogram (EEG)—when we move or observe movement^33^. Moreover, sensory-motor engagement impacts the appreciation of artworks. For example, participants that were asked to contract their facial muscles while looking at a painting with a painful expression gave higher artistic beauty ratings compared to participants who were asked to refrain from making any facial movements^34^. Our experience of art seems to be deeply linked to the way the artist engages with the world: by mentally reconstructing the creative process behind an artwork the viewer generates a new, interactive and dynamic experience^35,36^. Collectively, the studies reported so far indicate that artworks are perceived in an embodied manner.

One of the key implications of extending embodied simulation theory to art experiences is that perceiving art involves the same simulation mechanisms that shape our everyday interactions with the world. However, the question of whether art experiences are intrinsically different compared to everyday experiences is still an open question in empirical aesthetics^37^ and the object of a lively theoretical debate. While some researchers sustain that art is special and intrinsically different from everyday experiences^37–40^others argue that those differences are mainly due to socio economics contextual factors rather than unique brain activations during art engagement^41^.

Consider a broken glass: in a hardware store a broken glass is seen as a dangerous waste, due to the fact that it can no longer afford its original purpose as a reflective surface or functional window. In her solo exhibition at the Tate Modern, Yoko Ono’s A HOLE^42^ placed a pane of glass deliberately shot through with a bullet in the middle of the exhibition space, inviting the viewers to ‘go to the other side of the glass and see through the hole’. In the museum context, Ono transforms the glass’s functional flaw into a symbol: an opportunity to explore themes of violence, loss, and renewal. This example illustrates how recontextualising an object within a museum setting can not only reshape its perception as art, but can also transform its affordances—and the ways that we embody it.

The prior knowledge and expectations that individuals have about art is often referred to as art schema^43,44^. This schema is activated when individuals believe that they are engaging with art (e.g., in a museum), which subsequently influences both their cognitive and emotional processing of the experience (e.g. the way they will engage with broken glass). Prinz^40^ proposes that what sets engagement with art apart from interaction with everyday objects is the adoption of an aesthetic stance that encourages the observer to look beyond the object’s practical or ordinary function (e.g., a broken reflective surface or window), affording its metaphorical or unexpected meanings (e.g., an embodied symbol of artistic ideas). An object can afford the aesthetic stance either through its perceptual features like form and colour, or through external cues like contextual expectations and the artist’s intentions. Critically, an aesthetic response arises when something about the object, either in its form or context, invites viewers to interpret it beyond its practical function, opening up metaphorical or emotional meanings​^40^. Unlike everyday interactions with objects, which involve direct and active engagement, art often invites a stance of inaction and contemplation, captured by the concept of beholding affordances^37^. While the idea of viewing objects beyond their practical functions echoes Kant’s idea of disinterested interest^45^ in the present study we refer to the modern conceptualisation of disinterestedness, which is characterised by a lack of emotional or bodily involvement towards practical ends^37^. In the example of A HOLE^42^ Ono’s recontextualisation of the broken glass in a museum environment shapes our embodied and motor responses to that object by creating opportunities for contemplation (e.g., looking through the hole) rather than physical interaction (e.g., disposing of it as waste). To our knowledge, no empirical study has examined whether bodily sensations associated with everyday objects differ depending on whether they occur in an art context or in everyday life.

The present study adopts an embodied cognition perspective to investigate how the embodied experience of everyday objects that afford specific activities (e.g., a bottle affording drinking) and their associated body activations is modulated by their depiction as either ready-made artworks or non-art images (image type, within participants variable: art vs. not-art), and by contextual information (condition, between participants variable: Museum, Commercial and Mixed). For detailed description of the mixed-design, see ‘Experimental Procedure’ in the ‘Methods’ section. Specifically, we provided three different contexts: in the Museum condition, everyday objects were presented as works of art; in the Commercial condition, the same objects were introduced as items photographed for sale; while in the Mixed condition, both contexts are presented, and participants are asked to guess which objects were artworks and which were products for sale. This contextual manipulation was intended to evoke distinct expectations in participants, prompting them to adopt a Museum, Commercial, or Mixed cognitive stance. In the context of this study, the Commercial condition should be considered as eliciting a more functional cognitive framing compared to the Museum condition, rather than as a purely functional or neutral framing itself. The Mixed condition, in which items were described as ‘made by professional artists or by people for sale online’, was included to determine whether the observed effects stemmed specifically from an emphasis on functional use compared to aesthetic appreciation linked to an artistic context. We address the following research questions: (a) Do experimentally-induced expectations (i.e., Museum vs. Commercial vs. Mixed stances) have an impact on bodily sensations reported when viewing everyday objects? (b) Does the image type (Art vs. Not-Art) moderate these bodily sensations? (c) When expectations are mixed (Mixed condition), is it possible to differentiate between Art and Not-Art image types? (d) Are distinct patterns of bodily sensation associated more with experimentally-induced expectations or with the image type?

We hypothesise that when Not-Art images of everyday objects are presented in a Commercial context, they will elicit more sensations in body parts linked to their functional use (e.g., a cup will activate the hands and lips) compared to art images. In contrast, in a Museum context, the same Not-Art images will evoke sensations in atypical body regions (e.g., a cup will elicit sensations in the forehead or knees), but these sensations may be weaker or less differentiated than those elicited by Art images. Finally, we expect that, regardless of the experimental context, Art images will activate fewer body parts associated with an object’s typical functionality compared to Not-Art images.

## Results

### Developing a new researchers-friendly BSM visualisation tool and analysis approach

Leveraging methodologies by Schino et al.^26^ Ruta and Schino^46^ and Nummenmaa et al.^19^ we developed a custom open-access R script to visualise BSMs, offering a user-friendly alternative to existing MATLAB-based methods^19^. Our new R script automates data preprocessing and enhances the visual representation of click density distributions, enabling standardised and reproducible visualisations of activations and deactivations across body areas. Additionally, it includes a loop function that allows custom filtering of BSMs by variables such as participant or stimuli, facilitating flexible and scalable data analysis. Full methodological details—including preprocessing steps, visualisation techniques, and R code—are provided in the Supplementary Information and freely accessible on OSF at https://osf.io/bke9q/?view_only=5d9f2c577d3f417db1abc1486e7cb776.

### Assessing the impact of condition and image type across body regions at individual level

To quantify bodily sensations, we computed an Embodiment Score based on self-reported clicks on the BSMs. For each participant, activation and deactivation click frequencies were summarised separately for each image across five body regions: Head, Chest, Abdomen, Upper Limbs, and Lower Limbs. To obtain the final Embodiment Score, we first calculated the difference between activation and deactivation click counts. Taking into consideration that this difference could be either a positive or negative value ranging from − 10 to 10, we applied a signed log transformation to normalise the distribution while preserving directional effects and reduce the impact of individual differences in click frequency:

$$log(1+|x|)\times\,sign(x).$$

We fitted a linear mixed-effects model (estimated using ML and BOBYQA optimiser) to assess the effect that Condition (Mixed vs. Commercial vs. Museum), Image Type (Art vs. Not-Art) and Body Part (Head vs. Chest vs. Abdomen vs. Upper Limbs vs. Lower Limbs), and the interactions between these factors have on Embodiment Score. The model included participants and image identity as random effects. The model’s total explanatory power was weak (conditional *R*2 = 0.08), with the fixed effects alone accounting for 6% of the variance (marginal *R*2 = 0.06). The model’s intercept, corresponding to Condition = Mixed, Image Type = Not-Art, and Body Part = Head, was at 0.31 (95% CI [0.26, 0.36], t(45017) = 13.12, *p* < .001).

Within this model, Embodiment Scores were significantly lower in the Chest, Abdomen, Upper Limbs and Lower Limbs than they were in the Head region (all *p* < 0.001), suggesting a general tendency for participants to report stronger bodily sensations in the Head region, regardless of Condition or Image Type (see Table S2 in the Supplementary Information).

We also found that Image Type (Art) had a significant and positive effect (β = 0.07, 95% CI [0.02, 0.13], t(45017) = 2.61, *p* = .009; Std. β = 0.09, 95% CI [0.02, 0.16]), indicating that Art images elicited higher Embodiment Scores overall than Non-Art images (MArt = 0.0994; MNot-art = 0.0913), regardless of Condition or Body Part.

**Fig. 1:**
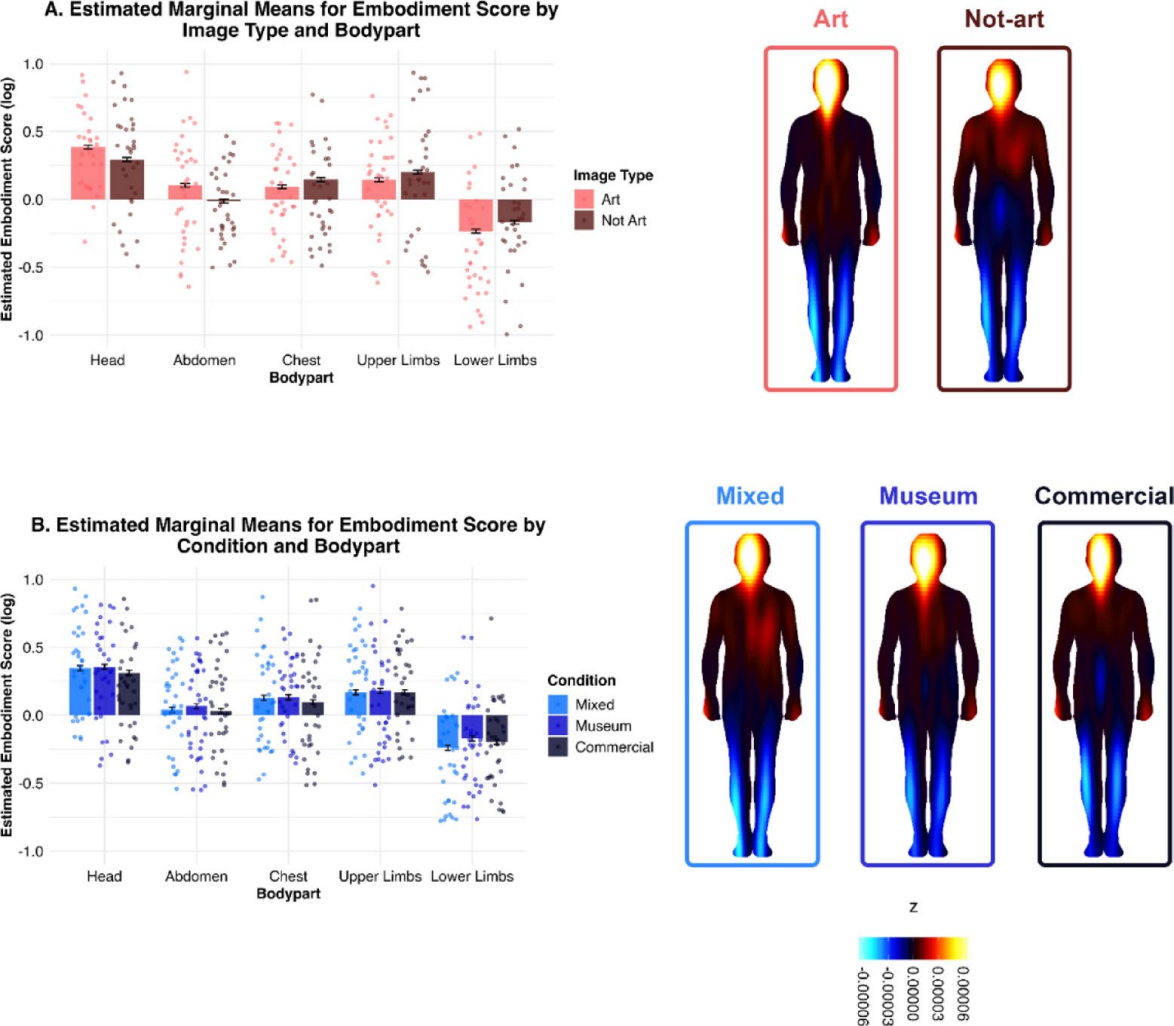
Bar plots (left) display the estimated Embodiment Score (log-transformed) from the mixed-effects model for each body part, separated by Image Type (**A**) and Condition (**B**). Corresponding Bodily Sensation Maps (BSMs) are shown on the right, illustrating average sensation distributions across body silhouettes as a function of Image Type (top row) and Condition (bottom row).

The effect of Image Type was significantly moderated by Body Part, indicating that bodily sensations differed depending on both the region of the body and whether the image was from the Art or Not-Art subset. Specifically, Art images elicited significantly lower Embodiment Scores than Non-Art images in the Chest, Upper Limbs, and Lower Limbs compared to the Head region (see Fig. 1A and Table S2). Post-hoc pairwise comparisons further clarified the interaction between Image Type (Art vs. Non-Art) and Body Part, showing that the effect of Image Type varies significantly across body regions. Specifically, for the Head and Abdomen body regions, Not-Art images elicited significantly lower Embodiment Scores compared to Art images (Head [Not-Art vs. Art]: estimate = -0.0925, SE = 0.0158, z = − 5.87, *p* < .0001; Abdomen [Not-Art vs. Art]: estimate = -0.1169, SE = 0.0158, z = − 7.418, *p* < .0001). On the other hand, for the Chest, Upper Limbs and Lower Limbs regions, we found the opposite pattern of results, with Not-Art images eliciting significantly higher Embodiment Scores than Art images (Chest [Not-Art vs. Art]: estimate = 0.0534, SE = 0.0158, z = 3.388, *p* = .0007; Upper Limbs [Not-Art vs. Art]: estimate = 0.0556, SE = 0.0158, z = 3.530, *p* = .0004; Lower Limbs [Not-Art vs. Art]: estimate = 0.0649, SE = 4.117, z = 4.117, *p* < .0001). These post-hoc comparisons were based on estimated marginal means averaged across all levels of Condition (Museum, Commercial and Mixed).

We found that Condition did not have a significant main effect on Embodiment Scores, nor was this effect moderated by Image Type. This indicates that, overall, experimentally-induced expectations (i.e., Museum, Commercial or Mixed stances) did not significantly alter bodily sensations when participants viewed Art vs. Non-Art images of everyday objects. However, Condition significantly interacted with Body Part, specifically when comparing Embodiment Scores in the Head and Lower Limbs regions. Compared to the Head, both the Commercial (β = 0.11, 95% CI [0.04, 0.19], t(45017) = 2.87, *p* = .004; Std. β = 0.14, 95% CI [0.05, 0.24]) and Museum conditions (β = 0.09, 95% CI [0.02, 0.17], t(45017) = 2.42, *p* = .015; Std. beta = 0.12, 95% CI [0.02, 0.22]) were associated with higher Embodiment Scores in the Lower Limbs relative to the Mixed condition (see Fig. 1B and Table S2). Post-hoc pairwise comparisons confirmed that this effect was mainly driven by the difference between the Mixed and Museum conditions in the Lower Limbs region (M = − 0.07, SE = 0.03, z = − 2.67, *p* = .023, Bonferroni-corrected). These post-hoc comparisons were based on estimated marginal means averaged across both levels of Image Type (Art and Non-Art).

There were no other significant effects in the model, confirming there was no significant interaction between Image Type and Condition, nor a three-way interaction with Body Part (see Table S2 in the Supplementary Information). Differences in Embodiment Score according to Image Type and Condition respectively are illustrated in Fig. 1, alongside corresponding BSMs.

### Is it art or not art?

**Fig. 2:**
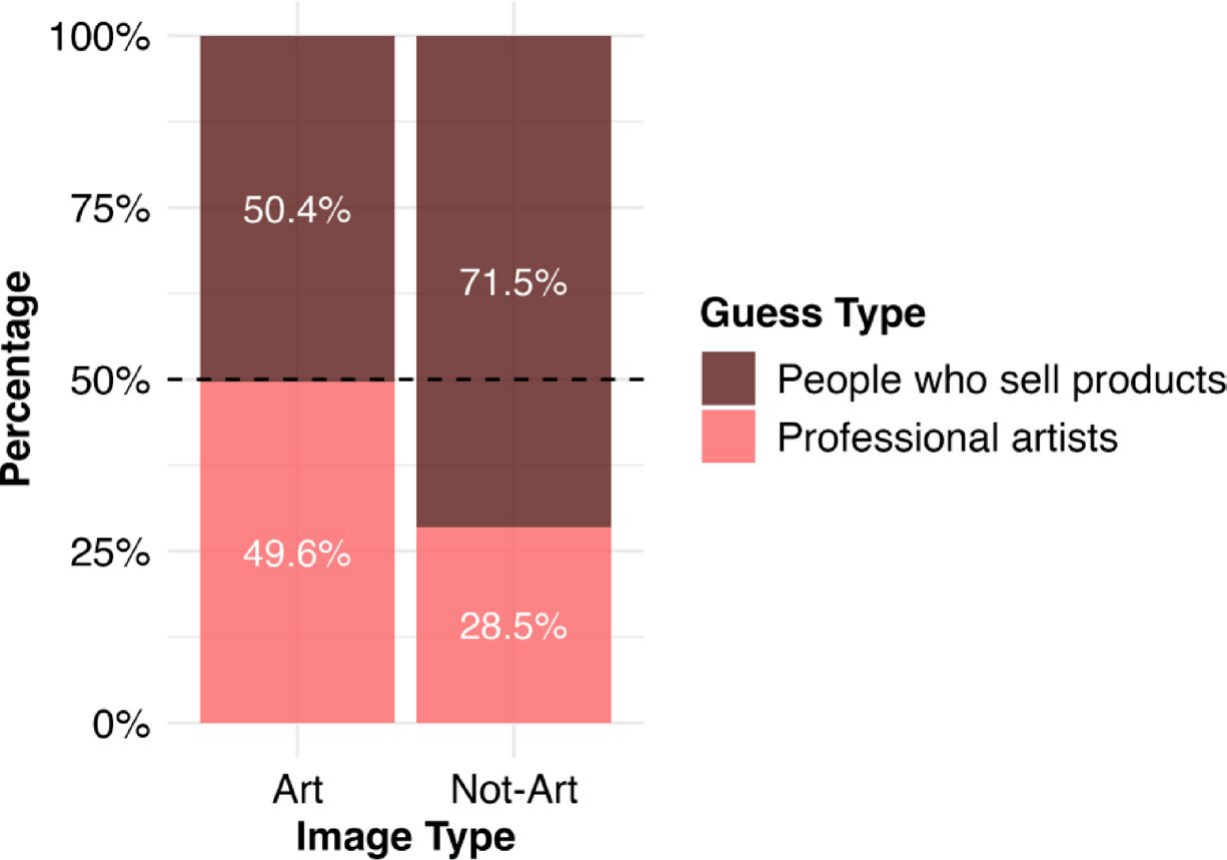
Stacked bar plot showing the percentage of guess response types by Image Type for participants in the Mixed condition only. Responses for Art images are shown in light red; responses for Not Art images are shown in dark red.

In the Mixed condition, we calculated the relative frequencies of participants’ guesses of whether they thought the images were made by professional artists or by people who sell products. When participants viewed images of everyday objects that were not artworks (Image Type: Not-art), they correctly identified them as images made by people selling items online in 71.49% of cases. However, when participants viewed images of ready made artworks (Image Type: Art), they correctly identified them as such in 49.62% of cases. A Pearson’s Chi-squared test with Yates’ continuity correction showed there was a significant association between the Image Type and participant’s guesses (χ^2^ (1, *N* = 85) = 143.74, *p* < .000), meaning that participants were significantly more likely to correctly identify Non-Art images as made by people who sell products than Art images as made by professional artists (Fig. 2).

Together, the findings reported so far show that both contextual framing (Condition) and whether an image depicts a ready-made artwork (Image Type) independently influence region-specific embodied responses (Body Part), but do not interact with each other. Specifically, the Mixed condition was associated with more polarised embodiment patterns between the Head and Lower Limbs, while Art images elicited higher Embodiment Scores than Not Art images in the Head and Abdomen. In the Mixed condition, where instructions did not invite participants to adopt a specific cognitive stance, participants were significantly better at identifying Not Art images than Art images, suggesting greater ambiguity in identifying artistic representations of everyday objects as artworks. Finally, the model revealed substantial variability in random effects associated with image identity, suggesting that embodiment responses may have been influenced by the everyday activities typically associated with the depicted objects (e.g., shirt with dressing, fork with eating). To account for this, we further explored whether different activities moderated BSMs embodiment patterns across Conditions and Image Type.

### Exploratory cluster analysis to assess the impact of specific activities at group level

We ran an exploratory analysis to investigate whether the colour patterns of the BSMs constituted clusters. We used the *patternize*^47^ *recolorize*^48^ and *colordistance*^49,50^ R package to generate a distance matrix of colour similarity for the BSMs across experimental condition and activity. This approach was inspired by the workflow proposed by Weller and the parameters selected by Schino et al.^51^ for the processing of BSMs data. Namely, the parameters are: standard red-green-blue (RGB) color space format, k-means clustering binning method, and Earth Mover’s Distance (EMD) as a measure of distance/dissimilarity^52^. The analysis aimed to group images with similar colour palettes, assessing whether the resulting clusters represented the most visually similar BSMs. Descriptive statistics for total number of pixels counted by body region, respectively associated with activations and deactivations colours, alongside Embodiment Score according to Regions of Interest, Condition and Image Type are reported in Table 1.

**Table 1:**
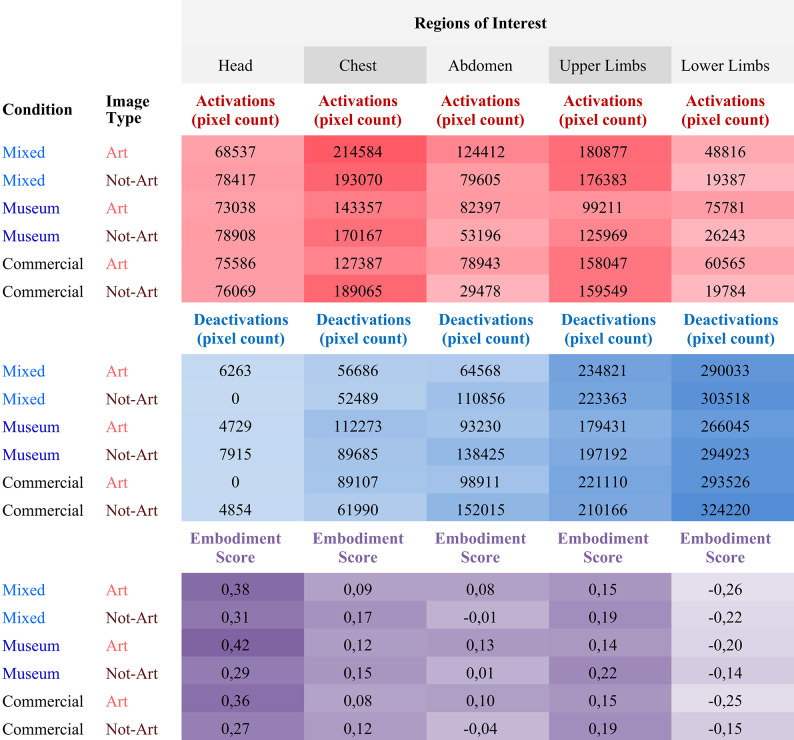
Descriptive statistics for three measures across regions of interest (head, chest, abdomen, upper limbs, and lower limbs), experimental conditions (mixed, museum, commercial), and image types (art, not art).

The first two measures—Activations (pixel count) and Deactivations (pixel count)—reflect the total number of pixels associated with activation (red) and deactivation (blue) areas in the BSMs, as identified by our custom image-processing code. The third measure, Embodiment Score (log-transformed), was derived from participants’ click responses and reflects the relative intensity of embodied sensations in each region. Activations are shown in shades of red, Deactivations in blue, Embodiment Score in purple, and regions of interest in grey.

Using a colour distance matrix generated with the *colordistance* R package (see Fig. S1 in Supplementary Information), we visualised pairwise similarity between BSM images as a heatmap. Lower distance values—represented by darker blue tones—indicate higher similarity between images, whereas higher distance values—represented by brighter pink tones—indicate lower similarity (or greater dissimilarity). To complement these results, we qualitatively inspected the hierarchical clustering of BSMs (see Fig. S2 in Supplementary Information), using the resulting dendrogram to explore potential recurring embodiment patterns and inform our diagnostic approach for the formal statistical analysis.

We observed that BSMs corresponding to the same activity in the Museum and Commercial conditions tend to show low colour distances, indicating a high degree of similarity in embodiment patterns across these two contexts. For example, the distance between the BSM generated for the Art image associated with the activity of dressing in Museum and Commercial conditions is very low (EMD ≅ 0.11; see Fig. S1). This suggests that, despite the different expectations induced by the two contextual information (Museum vs. Commercial), the same activity tends to evoke similar BSMs for Art images associated with the activity of dressing. Similarly, Not-Art images associated with the same activity result in BSMs that cluster close together.

**Fig. 3:**
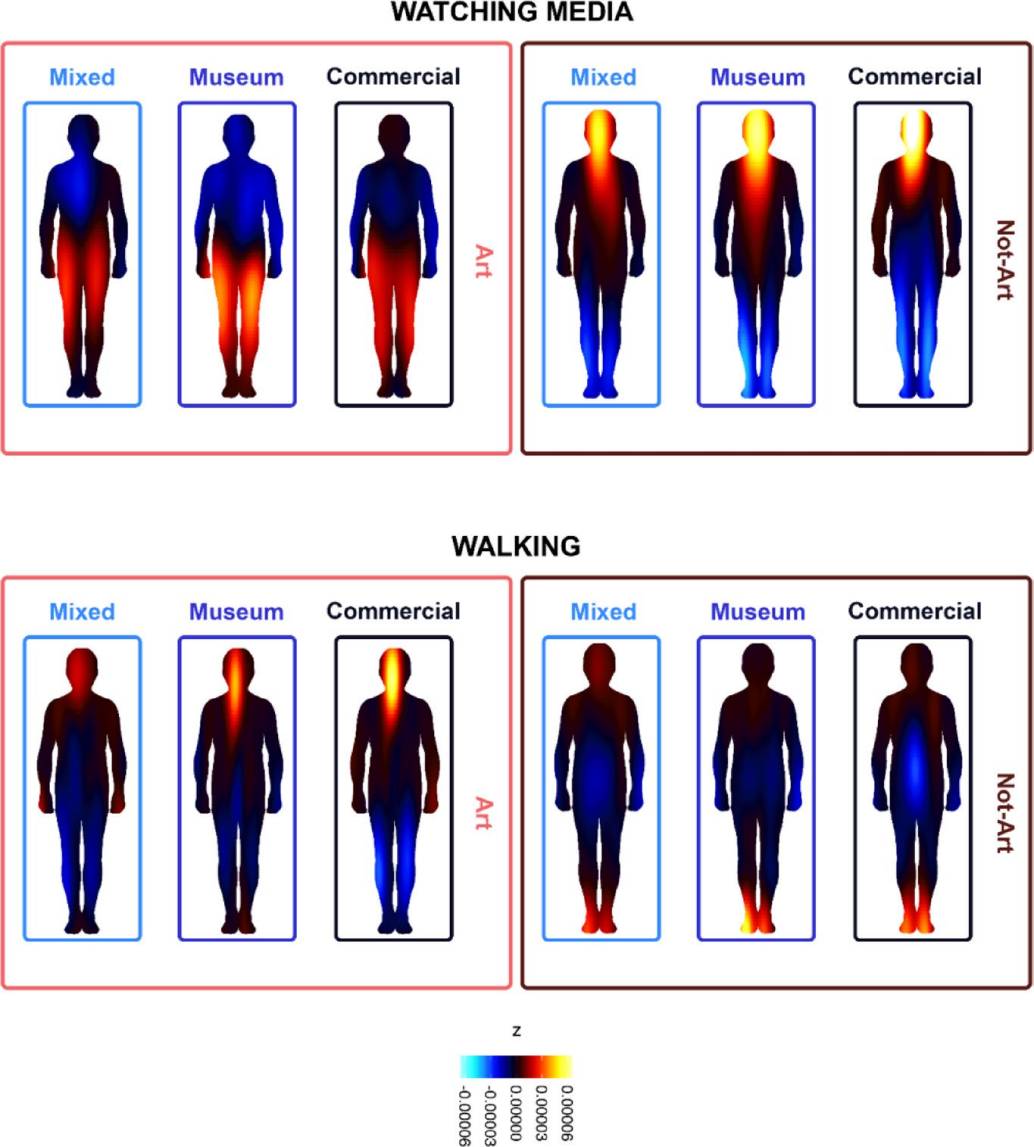
BSMs for the activities ‘Watching Media’ (top) and ‘Walking’ (bottom), shown across conditions (mixed, museum, commercial) and image types (art vs. not art).

However, when comparing Art and Not-Art images depicting objects associated with the same activity, we can observe a different pattern of results. As can be seen in Fig. 3, for activities such as ‘watching media’ and ‘walking’ the difference between BSMs varies depending on whether or not the image is Art or Not-Art. In these cases, the Image Type seems to be critical in inducing different patterns of bodily activity. For example, Art images depicting objects associated with ‘walking’ clearly evoked higher deactivations in the Lower Limbs region than Not-Art images did, in line with the results predicted by the models. Similarly, Art images connected to the activities of ‘watching media’ and ‘eating’ generated unique BSMs that were more distinct than those generated by Not-Art images associated with the same activities (see Fig. 3 and Fig. S2). To further investigate the moderating role of activity, we fitted an additional model including Activity as a fixed effect (full details of the model are available on OSF: ‘Exploratory Cluster Analysis And BSMs: Supporting Model – Activity’). Results from this model further corroborated the cluster analysis findings, showing that, across all conditions, head embodiment was significantly lower for Art compared to Not-Art images for the activity of ‘watching media’, and significantly higher for ‘walking’. Notably, BSMs generated in response to Art images related to activities like ‘reading’ in both the Museum and Commercial conditions consistently appeared distinct from BSMs for other activities. Art images depicting everyday objects associated with the activity of ‘reading’ in both the Museum and Commercial conditions generated BSMs that showed high dissimilarity scores (EMD > 0.35) compared to most other activities, indicating a unique bodily activation pattern for this activity and Image Type.

## Discussion

The present study is the first empirical investigation capturing affordances evoked by everyday objects through maps of self-reported bodily sensations. Our aim was to investigate how experimentally-induced expectations about the artistic status (Condition) of everyday objects influence self-reported embodiment activity, while accounting for whether the images were actually from art institutions or commercial websites (Image Type). Moreover, we outlined an innovative analytical approach that combines multiple quantitative analyses with qualitative inspection of BSMs to reveal nuanced, complex and richer patterns of bodily responses at both individual and group levels. This integrative strategy enabled a deeper and meaningful understanding of how contextual and perceptual factors shape embodiment responses to affordances evoked by everyday objects. One of the key contributions of the present study is our improved approach to BSMs analysis, which enabled us to unveil and better quantify colour pattern variations across relevant experimental variables. In line with Open Science principles, we decided to make the R code for visualising and analysing BSMs openly available to the research community. In doing so, we aim to enhance accessibility, transparency, and reproducibility of our findings to facilitate cross-study comparisons, and encourage broader adoption of BSM methods across disciplines.

When taking into account individual differences, our analysis of experimentally-induced expectations revealed that the difference in Embodiment Scores between the Head and Lower Limbs was significantly smaller in the Commercial and Museum conditions compared to the Mixed condition. Most notably, the Museum condition reported significantly less deactivation in the Lower Limbs than the Mixed condition did. When assessing the role of image type, results showed that Art and Not-Art images had a significant and clear impact on self-reported embodiment activity in different body regions regardless of the everyday object depicted. Specifically, Art images reported significantly more activity in the Head and Abdomen, as well as significantly less activity in Chest, Lower Limbs and Upper Limbs areas than Not-Art images did. Considering that our results did not reveal a significant interaction between contextual information (Mixed vs. Museum vs. Commercial) and image type (Art vs. Not-art) on Embodiment Score, and that participants were not more likely to recognise Art images as professionally produced, our findings suggest that the artistic depiction of everyday objects and viewers’ expectations each play important yet distinct roles in our embodied experience of art encounters.

When assessing group-level effects, cluster analysis on BSMs revealed that the nature of the image and the activity associated with the everyday objects depicted in it played a critical role in shaping embodiment patterns. For activities such as ‘watching media’, ‘working’, and ‘sleeping’, which typically involve a seated posture or minimal engagement of the Lower Limbs, participants reported less Lower Limb deactivation when viewing Art images than when viewing Not-Art images. To better understand these findings, we will take a closer look at the images associated with the activity of ‘watching media’. In our dataset, both image types depicted a TV screen— the Art image, the artwork entitled Gilbert & George’s *‘In the Bush’*^53^ showed a screen positioned on a tall plinth against a plain white wall, while the Not-Art image, an advertisement of a *Edgeware Small TV Unit* showed a flat TV screen on a low cabinet with storage shelves in a modern living-room interior. The BSM embodiment patterns for the ‘watching media’ Art image revealed higher activations at the level of the Lower Limbs than the Not-Art image did. Conversely, the Not-Art image elicited strong activations in the Head area that were absent from the Art image’s BSMs. There are two important insights to draw from these findings. First: the complex and nuanced pattern of results that emerged from the cluster analysis highlights how individual activities (like ‘watching media’) display characteristic activation patterns that are deviations from and sometimes opposite to the global trend. Second: the recurring clustering of BSM according to image type suggests that Art images depicting everyday objects may reduce sensations typically associated with the functional affordances of those objects and may elicit bodily responses distinct from Non-Art images depicting the same objects. As was the case for image type, we found that individual activities also played an important role in shaping BSM embodiment patterns for the different conditions. For example, when analysing an utilitarian activity like ‘eating’, image cluster analysis revealed that Not-Art images showed more activations at the level of the Head (i.e. mouth and throat areas) in the Commercial condition compared to the Museum and Mixed conditions. At the same time, Art images clustered together in a separate location, showing an increased activity both at the level of the Head and Abdomen across all conditions. Taken together these results demonstrate the need to assess BSMs in terms of activation patterns across the whole body to reveal more meaningful, richer and generalisable insights.

Taken together, our findings suggest that while artistic qualities of the image primarily modulate activity in specific body regions that are more or less typically associated with the functional use of everyday objects, contextual framing influences the relative intensity of embodied responses, underscoring the multifaceted nature of embodied art experiences. We can interpret these findings to indicate that images of ready-made artworks have been deliberately created by art professionals to disrupt the typical functional associations the viewer makes with everyday objects, therefore eliciting less functionally driven bodily sensations. Critically, the results showing diversified embodiment patterns for Art and Not-Art images aligns with predictions from embodied simulation theory^54^ which predicts that art can transform or subvert ordinary affordances by eliciting bodily responses that differ from those elicited by more functionally oriented depictions. We can therefore speculate that when engaging with ready-made artworks, we temporarily distance ourselves from everyday tasks, creating more space for our mental simulation processes to engage in less utilitarian affordances and bodily sensations. Overall, our pioneering approach to BSMs contributes to a better understanding of how context-driven expectations and images of ready-made art shape bodily responses.

The use of the BSMs has represented an interdisciplinary method of investigation, since body mapping was used for the exploration of all art forms by Violet Paget (‘Vernon Lee’) and Clementina Anstruther-Thomson during excursions at museums and galleries^55^. Paget and Anstruther-Thomson claimed that embodied experiences and bodily sensations are the factors that give rise to mental impressions of beauty or feelings of pleasure (not the other way around), echoing the principles of embodied cognition framework. We demonstrate that BSMs serve as more than visually appealing graphics; they are computationally efficient and enable rapid data processing. This enhances our ability to understand, explore, and compare art experiences holistically in both quantitative and qualitative terms. However, when data volume grows, the overplotting that results may lead to overlapping points obscuring underlying relationships. In such cases, the interpretability of results may diminish. Hence, the importance of our preprocessing approach that computes the differences between activations and deactivations to mitigate this effect.

While the findings offer valuable insights, certain limitations must be taken into account. First, a potential limitation of the present study is that we did not assess participants’ frequency of museum visits. Considering that familiarity with art contexts could potentially influence activations of both art and body schemata, it would be interesting to explicitly assess the impact of this variable in future research. Second, an additional limitation of our study is the potential stimulus set effect^56,57^. It is possible that our reliance on a specific set of everyday-object images, although sourced from both art institutions and commercial websites, might restrict the generalisability of our findings, as the limited variability in stimulus types could have influenced participants’ embodiment responses. Building on our findings, a potential direction for future research will be to disentangle stimuli set-related and context-related contributions to bodily activity by selecting stimuli specifically associated with localised body regions (e.g. head), first establishing their localized embodied responses, and then assessing how contextual framing might modulate these responses. Additionally, researchers interested in the emotional implications of affordances in ready-made art could build on this approach by systematically evaluating the emotional valence of everyday objects and examining its relationship with different contextual information, such as the ones activated by the art schema.

By revealing how subtle shifts in context and perception shape the way our bodies respond to everyday objects, this study not only advances our understanding of embodied aesthetics, but also lays the groundwork for a more reproducible and interdisciplinary science of felt experience.

## Methods

### Participants

All participants were recruited on Prolific (www.prolific.com*)*, a platform for online research. To ensure high quality data, the study had the following selection criteria: Participants had to (1) be fluent in English and (2) have at least an 80% success rate of experiment completion. The study was developed on Qualtrics, an online software for conducting psychological studies (www.qualtrics.com*).*

A total of 277 participants took part in the study. Twelve participants were excluded from the final analysis due to poor data quality (e.g., no clicks reported on body silhouettes) or failure to pass all attention checks, resulting in a final sample of 265 participants (*M*_*age*_ = 40.11, *SD* = 13.85; 89 women, 172 men, 2 non-binary and 2 preferred not to report their gender; exclusion criteria: approval rate > 80, UK based and English as first language ).

As recommended by Atari, Davani & Dehghani and Nummenmaa and colleagues, studies exploring self-reported bodily sensations should ideally recruit at least 40 participants per group to ensure adequate statistical power^19,21^. Similar studies conducted with the use of BSMs estimated approximately a similar number of participants taking part in the experiments^22,58^. Therefore, we aimed at having at least 40 participants per condition. Participants were randomly assigned to one of the three experimental Conditions: Museum (*n* = 92), Commercial (*n* = 93), and Mixed (*n* = 92). After exclusions, 89, 91, and 85 participants were retained in each group, respectively.

### Stimuli selection

We aimed to select a set of objects that people frequently associate with daily activities. To this end, we conducted a pilot study, recruiting 40 participants on Prolific (*M*_*age*_ = 42.05, *SD* = 13.66; 27 women and 13 men) who did not take part in the main study. Participants were asked to describe activities they perform on a daily basis and the objects they associate with these activities (e.g., drinking: cup). Overall, participants mentioned 25 activities and 536 objects (for the full list of activities, objects and their frequencies, please refer to Table S3 in the Supplementary Information). Based on the results, the most frequent activities were: eating, walking, sleeping, working, showering, dressing, watching media, drinking, reading, cleaning and observing. Previous studies^26,27^ found that when participants viewed visual art, their BSMs showed increased activation in the head region, a result interpreted as linked to the act of observing. Therefore, we decided to include objects associated with the activity of observation in our stimuli selection to help determine whether head-level activation is linked to contextual information (i.e., whether it is consistently reported across all BSMs regardless of the activity associated with the object depicted in the image or whether it varies according to the expectations shaped by the set of instructions given to the participant).

We then selected the objects most commonly associated with specific everyday activities, such as a shirt for dressing and a coffee cup for drinking. For each activity, we selected pairs of images depicting the same object: one sourced from art institution websites (Image Type: Art) and the other from popular commercial sites that sell new and used goods online, such as eBay (Image Type: Not-Art). The final image set consisted of 68 images in total, representing 34 objects, each depicted by both Art and Not-Art image type. We provide the full list of images, including the depicted objects, corresponding activity, image type (Art vs. Not-Art) and website source in Table S1 in the Supplementary Information.

### **Experimental procedure**

**Fig. 4:**
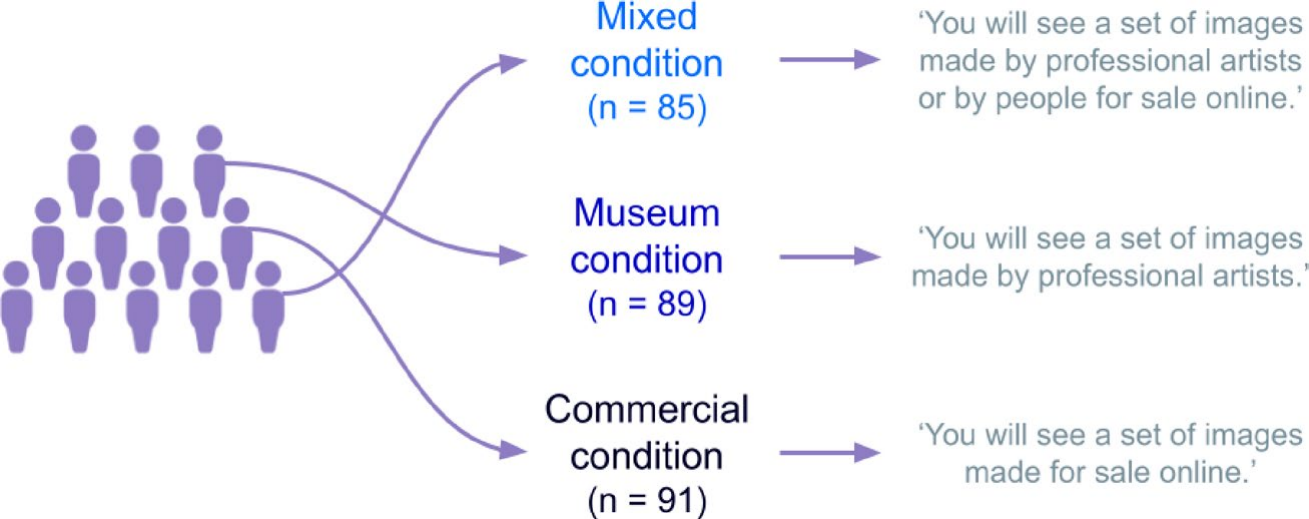
Diagram showing experimental condition allocation.

We used a mixed-design, where participants were randomly assigned to one of the three experimental conditions: Museum, Commercial, or Mixed (see Fig. 4). Each condition was designed to induce a specific cognitive stance by providing participants with a distinct set of instructions. The instructions were presented at the beginning of the study, with the aim of shaping participants’ expectations regarding the images they would see during the course of the experiment (see Table S4 in the Supplementary Information for the original instructions provided in each experimental condition).

**Fig. 5:**
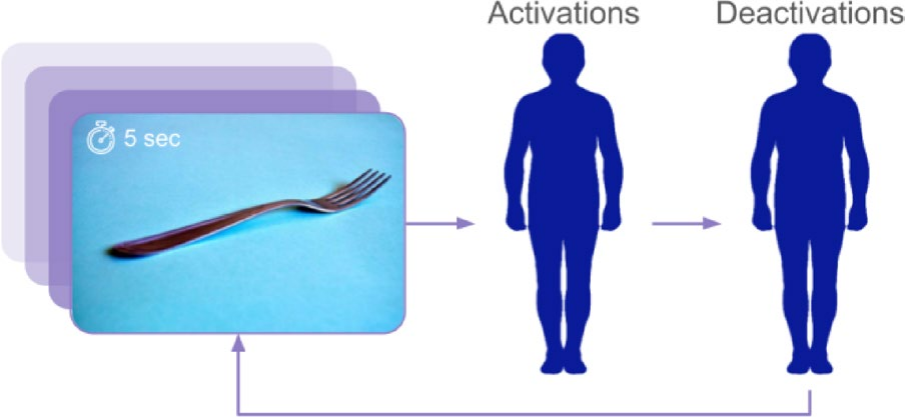
Diagram showing trial procedure for each image.

Immediately after receiving the instructions, the trials began (see Fig. 5). Each image was displayed on-screen for 5 s, accompanied by the following text: ‘Look at this picture and think carefully about your body sensations while viewing it.’ Each participant saw 34 images in total, with objects counterbalanced by image type (17 Art and 17 Non-Art) and images presented in a random order. The design ensured that each participant saw all 34 objects, and avoided presenting both image types of the same object.

Participants were then asked to report their bodily sensations by using two body silhouettes: one for activations (stronger sensations, more energy) and another for deactivations (weaker sensations, less energy). There was no time limit for this task. Participants were informed that they could click up to 10 times and were encouraged to use multiple clicks to emphasise more significant activation or deactivation in a specific body area (see Fig. 5).

After seeing each image and reporting their bodily sensations, only participants in the Mixed condition were additionally asked to assess who they thought created the image. They could choose between two options: (1) professional artists or (2) people who sell products.

## Supplementary Information

Below is the link to the electronic supplementary material.

Supplementary Material 1

## Data Availability

Due to copyright and availability restrictions, the links to the specific images and advertisement images used as stimuli in this study cannot be publicly shared. However, we provide the full list of images selected for the study, relevant image information and the related source link in Table S1. Additionally, the full image database will be shared for research purposes by contacting directly the corresponding authors. The data and analysis code that support the findings of this study are openly available at: https://osf.io/bke9q/?view_only=5d9f2c577d3f417db1abc1486e7cb776.

## References

[1] Chatterjee, A. The neuropsychology of visual artistic production. *Neuropsychologia* **42**, 1568–1583 (2004). 15246293 10.1016/j.neuropsychologia.2004.03.011

[2] Freedberg, D. & Gallese, V. Motion, emotion and empathy in esthetic experience. *Trends Cogn. Sci.* **11**, 197–203 (2007). 17347026 10.1016/j.tics.2007.02.003

[3] Breton, A. & Duchamp, M. *Le Surréalisme en 1947*. (Pierre à Feu, Maeght Éditeur, Paris, France, (1947).

[4] Duchamp, M. *Bicycle Wheel*. (1913).

[5] Picasso, P. *Bull’s Head*. (1942).

[6] Burnett, M. & Gallagher, S. 4E cognition and the spectrum of aesthetic experience. *Jolma J*. 10.30687/Jolma/2723-9640/2020/02/001 (2020).

[7] Keijzer, F. A. *Representation and Behavior* (MIT Press, 2001).

[8] Merleau-Ponty, M. *The World of Perception* (Routledge, 1948).

[9] Dewey, J. *Art as Experience*362 (Minton, Balch, 1934).

[10] Hildebrand, A. *The Problem of Form in Painting and Sculpture* (G. E. Stechert & Co., 1907).

[11] Loren, L. A. & Dietrich, E. Merleau-ponty embodied cognition, and the problem of intentionality. *Cybernetics Syst.* **28**, 345–358 (1997).

[12] Gibson, J. J. *The Ecological Approach To Visual Perception: Classic Edition* (Psychology, 1979).

[13] Rietveld, E. The affordances of Art for making technologies. *Adapt. Behav.* **30**, 489–503 (2022). 36404908 10.1177/10597123221132898 PMC9667099

[14] Gallese, V., Fadiga, L., Fogassi, L. & Rizzolatti, G. Action recognition in the premotor cortex. *Brain* **119**, 593–609 (1996). 8800951 10.1093/brain/119.2.593

[15] Rizzolatti, G. & Craighero, L. The mirror-neuron system. *Annu. Rev. Neurosci.* **27**, 169–192 (2004). 15217330 10.1146/annurev.neuro.27.070203.144230

[16] Gallese, V. The empathic body in experimental Aesthetics – Embodied simulation and art. in Empathy (eds Lux, V. & Weigel, S.) 181–199 (Palgrave Macmillan UK, London, doi:10.1057/978-1-137-51299-4_7. (2017).

[17] Gallese, V., Keysers, C. & Rizzolatti, G. A unifying view of the basis of social cognition. *Trends Cogn. Sci.* **8**, 396–403 (2004). 15350240 10.1016/j.tics.2004.07.002

[18] Clark, A. An embodied cognitive science? *Trends Cogn. Sci.* **3**, 345–351 (1999). 10461197 10.1016/s1364-6613(99)01361-3

[19] Nummenmaa, L., Glerean, E., Hari, R. & Hietanen, J. K. Bodily maps of emotions. *Proc. Natl. Acad. Sci. USA*. **111**, 646–651 (2014). 24379370 10.1073/pnas.1321664111 PMC3896150

[20] Nummenmaa, L., Hari, R., Hietanen, J. K. & Glerean, E. Maps of subjective feelings. *Proc. Natl. Acad. Sci. USA*. **115**, 9198–9203 (2018). 30154159 10.1073/pnas.1807390115 PMC6140475

[21] Atari, M., Mostafazadeh Davani, A. & Dehghani, M. Body maps of moral concerns. *Psychol. Sci.* **31**, 160–169 (2020). 31913779 10.1177/0956797619895284

[22] Novembre, G., Zanon, M., Morrison, I. & Ambron, E. Bodily sensations in social scenarios: where in the body? *PLoS One*. **14**, e0206270 (2019). 31185013 10.1371/journal.pone.0206270 PMC6559636

[23] Rinne, P., Tavast, M., Glerean, E. & Sams, M. Body maps of loves. *Philosophical Psychol.* 1–23. 10.1080/09515089.2023.2252464 (2023).

[24] Putkinen, V. et al. Bodily maps of musical sensations across cultures. *Proc. Nati. Acad. Sci.* **121**, e2308859121 (2024). 10.1073/pnas.2308859121 PMC10835118 38271338

[25] García-Magariño, I., Chittaro, L. & Plaza, I. Bodily sensation maps: exploring a new direction for detecting emotions from user self-reported data. *Int. J. Hum. Comput. Stud.* **113**, 32–47 (2018).

[26] Schino, G., Van Klaveren, L.-M., Gallegos González, H. G. & Cox, R. F. A. Applying bodily sensation maps to art-elicited emotions: an explorative study. *Psychol. Aesthet. Creativity Arts*. **18**, 315–329 (2021).

[27] Nummenmaa, L. & Hari, R. Bodily feelings and aesthetic experience of art. *Cogn. Emot.* **37**, 515–528 (2023). 36912601 10.1080/02699931.2023.2183180

[28] Provenzano, L. et al. Embodiment of underweight and normal-weight avatars affects bodily self-representations in anorexia nervosa. *Heliyon* **10**, e32834 (2024). 38988549 10.1016/j.heliyon.2024.e32834 PMC11233954

[29] Marucci, M. et al. Rewiring the evolution of the human hand: how the embodiment of a virtual bionic tool improves behavior. *iScience* **27**, 109937 (2024). 39055602 10.1016/j.isci.2024.109937 PMC11270032

[30] Gallagher, S. How the body shapes the Mind. *How Body Shapes Mind*. **1–294**10.1093/0199271941.001.0001 (2006).

[31] de Vignemont, F. Body schema and body image–pros and cons. *Neuropsychologia* **48**, 669–680 (2010). 19786038 10.1016/j.neuropsychologia.2009.09.022

[32] Schema *APA Dictionary of Psychology* (2018).

[33] Umiltà, M. A., Berchio, C., Sestito, M., Freedberg, D. & Gallese, V. Abstract art and cortical motor activation: an EEG study. *Front Hum. Neurosci* **6**, 311 (2012). 10.3389/fnhum.2012.00311 PMC3499799 23162456

[34] Ardizzi, M. et al. Beholders’ sensorimotor engagement enhances aesthetic rating of pictorial facial expressions of pain. *Psychol. Res.* **84**, 370–379 (2020). 30073408 10.1007/s00426-018-1067-7

[35] Brinck, I. Empathy, engagement, entrainment: the interaction dynamics of aesthetic experience. *Cogn. Process.* **19**, 201–213 (2018). 28391411 10.1007/s10339-017-0805-x PMC5976699

[36] Cox, R. F. A. & van Klaveren, L. M. The embodied experience of abstract art: an exploratory study. *Ecol. Psychol.* **36**, 111–122 (2024).

[37] Brincker, M. The aesthetic Stance—on the conditions and consequences of becoming a beholder. in Aesthetics and the Embodied Mind: Beyond Art Theory and the Cartesian Mind-Body Dichotomy (ed Scarinzi, A.) vol. 73 117–138 (Springer Netherlands, Dordrecht, (2015).

[38] Cupchik, G. C. The evolution of psychical distance as an aesthetic concept. *Cult. Psychol.* **8**, 155–187 (2002).

[39] Menninghaus, W. et al. The distancing-embracing model of the enjoyment of negative emotions in art reception. *Behav. Brain Sci.* **40**, e347 (2017). 28215214 10.1017/S0140525X17000309

[40] Prinz, J. Emotion and aesthetic value. in The Aesthetic Mind: Philosophy and Psychology (eds Schellekens, E. & Goldie, P.) 0 (Oxford University Press}. 10.1093/acprof:oso/9780199691517.003.0006. (2011).

[41] Skov, M. & Nadal, M. A farewell to art: aesthetics as a topic in psychology and neuroscience. *Perspect. Psychol. Sci.* **15**, 630–642 (2020). 32027577 10.1177/1745691619897963

[42] Ono, Y. *A HOLE*. (2009).

[43] Jacobsen, T. Bridging the arts and sciences: a framework for the psychology of aesthetics. *Leonardo* **39**, 155–162 (2006).

[44] Wagner, V., Menninghaus, W., Hanich, J. & Jacobsen, T. Art schema effects on affective experience: the case of disgusting images. *Psychol. Aesthet. Creativity Arts*. **8**, 120–129 (2014).

[45] Kant, I. *Critique of the Power of Judgment*. (Cambridge University Press, Cambridge, 1790). 10.1017/CBO9780511804656

[46] Ruta, N. & Schino, G. Bodily Sensation Maps to capture valence and arousal of artistic images: developing a new methodology. (2023).

[47] van Belleghem, S. Quantification of color pattern variation. 0.0.5 (2017). 10.32614/CRAN.package.patternize

[48] Weller, H. *Color-Based Image Segmentation 0.1.0*. 10.32614/CRAN.package.recolorize (2021).

[49] Weller, H. colordistance: Distance Metrics for Image Color Similarity. (2021).

[50] Weller, H. & Westneat, M. Quantitative color profiling of digital images with earth mover’s distance using the R package colordistance. *PeerJ* **7**, e6398 (2019). 30775177 10.7717/peerj.6398 PMC6371918

[51] Schino, G., van Klaveren, L. M., van Dorsten, T., Van Heusden, B. & Cox, R. F. A. Art Is in The Body of The Beholder: Examining Emotions in Children and Adolescents’ Art Experiences. (2025).

[52] Rubner, Y., Tomasi, C. & Guibas, L. J. The Earth mover’s distance as a metric for image retrieval. *Int. J. Comput. Vision*. **40**, 99–121 (2000).

[53] Gilbert, P. & George, P. *In the Bush*. (1972).

[54] Gallese, V. Naturalizing aesthetic experience: the role of (Liberated) embodied simulation. (2018). 10.3167/proj.2018.120207

[55] Lee, V. *The Psychology of an Art Writer* (David Zwirner Books, 1903).

[56] Temme, J. E. Effects of Mere exposure, cognitive set and task expectations on aesthetic appreciation. in *Advances in Psychology* (eds Crozier, W. R. & Chapman, A. J.) vol. 19 389–410 (North-Holland, (1984).

[57] Bilalić, M., McLeod, P. & Gobet, F. The mechanism of the einstellung (Set) effect: a pervasive source of cognitive bias. *Curr. Dir. Psychol. Sci.* **19**, 111–115 (2010).

[58] Torregrossa, L. J. et al. Anomalous bodily maps of emotions in schizophrenia. *Schizophr Bull.* **45**, 1060–1067 (2019). 30551180 10.1093/schbul/sby179 PMC6737484

