# Supplementary material for: Ready-made bodily sensations
Source: Sci Rep. 2025 Aug 18;15:30223. doi: 10.1038/s41598-025-14061-5 (PMC12361478; doi:10.1038/s41598-025-14061-5)
Supplement: Supplementary file 1 — Supplementary Material 1 [file 41598_2025_14061_MOESM1_ESM.pdf]

Supplementary Information
Ready-Made Bodily Sensations

Nicole Ruta[1,+*], **Gemma Schino**[2,+], **Marina Iosifyan**[1], **and Brendan Wolfe**[1]

[1]School of Divinity, University of St Andrews, St Mary's College, St Andrews, KY16 9JU, Scotland
[2]Department of Psychology, University of Groningen, Groningen, 9712 TS, The Netherlands
[*]nvgr1@st-andrews.ac.uk
[+]these authors contributed equally to this work

Author's Note

Nicole Ruta https://orcid.org/0000-0001-5300-2913

Gemma Schino https://orcid.rg/0000-0001-5757-6359

Marina Iosifyan https://orcid.org/0000-0002-6617-5116

Brendan Wolfe https://orcid.org/0000-0001-8848-7244

Supplementary Information Guide:

Supplementary Notes

Detailed notes on data pre-processing and introduction to custom code for Bodily Sensations Maps (BSMs) visualisation

Supplementary Figures

Supplementary figures from S1 to S2

Supplementary Tables

Supplementary tables S1 to S4

Supplementary References

List of supplementary references of additional literature mentioned in the supplementary notes

Supplementary Notes

Data pre-processing and introduction to custom code for Bodily Sensations Maps (BSMs) visualisation

For this project, we collected *xy*-coordinates from the points representing perceived sensations (activations and deactivations) that participants placed on the blank body silhouettes. Leveraging methodologies by Schino et al. [1] and Nummenmaa et al. [2] and with the aim of providing an accessible, open-source alternative to the MATLAB approach used by Nummenmaa et al. [2], we developed an R script to streamline the preprocessing of these data points and enhance the visual representation of density distributions (Ruta & Schino, 2023). The R script developed for the present research improves on the Excel-based method outlined by Schino et al. [1] and is freely accessible on OSF (see Additional information in the main text). The three-step process was preceded by the raw data being downloaded in CSV format from Qualtrics and then uploaded to RStudio (RStudio Team, 2020; R version 4.4.1).

The process began with the application of the 2D kernel density estimation function from the *MASS* R package [4]. This function, adapted from Slowikowski's code [5], produces raw density measures that serve as a foundation for visual analysis. We then applied a tailored colour scheme inspired by Rennie's palette customisation approach [6]. This allows us to improve the visual identity, to differentiate areas of varied density and to incorporate the same colours used in previous BSMs studies for consistency and standardisation [7].

The second customised function, adapted from Mallepalle et al. [8] and Mock's work [9], enables us to estimate and compare densities across the silhouette. We processed the data to fit a polygon around high-density areas (where frequent participant markings coalesced) and colour-coded density values to reflect the concentration of clicks. The *xy*-coordinates define the position on the body, while the *z* variable denotes the density of markings in each area.

In the third and final step, we generated two separate density estimates for "activations" and "deactivations", subtracting the deactivation density from activation density to create a nuanced dataset. This allows us to visualise embodiment responses in contrasting regions, resulting in a visually intuitive output suitable for further analysis and plotting. The BSMs generated can yield both qualitative (via visual inspection) as well as quantitative results (via frequency counts, omnibus and non-parametric test outputs, cluster analyses, and image-based analysis).

In this study, we also developed a second custom R script to identify specific body regions on the human silhouette (Head, Chest, Abdomen, Upper Limbs and Lower Limbs) and to count the number of pixels that matched the predefined colors used to generate activation and deactivation areas in the BSM images. We used the *imager* package for image processing [10], *dplyr* for data manipulation [11], and *writexl* to export the results to an Excel file [12].

The code for the process described above is freely accessible on OSF (see 'BSM Visualisations 2025.R' for visualising BSMs and 'BSM ROI Finder 2025.R' for identifying specific body regions and counting coloured pixels).

Supplementary References

1. Schino, G., Van Klaveren, L.-M., Gallegos González, H. G. & Cox, R. F. A. Applying bodily sensation maps to art-elicited emotions: An explorative study. *Psychol. Aesthet. Creat. Arts* **18**, 315–329 (2021).
2. Nummenmaa, L., Glerean, E., Hari, R. & Hietanen, J. K. Bodily maps of emotions. *Proc. Natl. Acad. Sci.* **111**, 646–651 (2014).
3. RStudio Team. RStudio. RStudio: Integrated Development for R. RStudio (2020).
4. Ripley, B. & Venables, B. MASS: Support Functions and Datasets for Venables and Ripley's MASS. 7.3-61 https://doi.org/10.32614/CRAN.package.MASS (2009).
5. Slowikowski, K. Color points by density with ggplot2. *Kamil Slowikowski - Notes* https://slowkow.com/notes/ggplot2-color-by-density/ (2017).
6. Rennie, N. Custom colour palettes for {ggplot2}. *Jumping Rivers* https://www.jumpingrivers.com/blog/custom-colour-palettes-for-ggplot2/ (2022).
7. García-Magariño, I., Chittaro, L. & Plaza, I. Bodily sensation maps: Exploring a new direction for detecting emotions from user self-reported data. *Int. J. Hum.-Comput. Stud.* **113**, 32–47 (2018).
8. Mallepalle, S., Yurko, R., Pelechrinis, K. & Ventura, S. L. Extracting NFL tracking data from images to evaluate quarterbacks and pass defenses. *J. Quant. Anal. Sports* **16**, 95–120 (2020).
9. Mock, T. Heatmaps in ggplot2. *The MockUp* https://themockup.blog/posts/2020-08-28-heatmaps-in-ggplot2/ (2020).
10. Barthelme, S. imager: Image Processing Library Based on 'CImg'. 1.0.2 https://doi.org/10.32614/CRAN.package.imager (2015).
11. Wickham, H., François, R., Henry, L., Müller, K. & Vaughan, D. dplyr: A Grammar of Data Manipulation. 1.1.4 https://doi.org/10.32614/CRAN.package.dplyr (2014).
12. Ooms, J. writexl: Export Data Frames to Excel 'xlsx' Format. 1.5.1 https://doi.org/10.32614/CRAN.package.writexl (2017).

Supplementary Tables

Object Depicted	Description	Retrieved From	Activity	Image Type
Toilet brush	Philippe Starck (1993), *The Excalibur Toilet Brush*.	moma.org	Cleaning	Art
Toilet brush	Online selling ad	gts-shop.co.uk	Cleaning	Not-Art
Vacuum cleaner	Matt Keegan (2016), *Have you seen my language?*	moma.org	Cleaning	Art
Vacuum cleaner	Online selling ad	expertreviews.co.uk	Cleaning	Not-Art
Belt	Matt Keegan (2016), *Have you seen my language?*	moma.org	Dressing	Art
Belt	Online selling ad	ebay.co.uk	Dressing	Not-Art
Shirt	Rodney Graham (1993), *White Shirt (for Mallarmé)*	tate.org.uk	Dressing	Art
Shirt	Online selling ad	dimaclasse.com	Dressing	Not-Art
T-shirt	Hermann Nitsch (1990), *Painted Shirt*	moma.org	Dressing	Art
T-shirt	Online selling ad	cooshti.com	Dressing	Not-Art
Jeans	Wolfgang Tillmans (1991), *Grey jeans over stair post*	www.guggenheim.org	Dressing	Art
Jeans	AI creative suite	freepik.com	Dressing	Not-Art
Coffee cup	Stephen Shore (1972), *Granite, Oklahoma*	tate.org.uk	Drinking	Art
Coffee cup	Online hardware development community	hackaday.com	Drinking	Not-Art
Teabag	Adolf Rambold (1949), *Double Chamber Tea Bag*	moma.org	Drinking	Art
Teabag	Online dictionary	dictionary.cambridge.org	Drinking	Not-Art
CocaCola	Cildo Meireles (1971), *Coca-Cola project*	tate.org.uk	Drinking	Art
CocaCola	Online selling ad	amazon.co.uk	Drinking	Not-Art
Milk	Harold Eugene Edgerton (1957), *Milk Drop*	mitmuseum.mit.edu	Drinking	Art
Milk	Dairy products online	arlafoods.co.uk	Drinking	Not-Art
Bread	Matt Keegan (2016), *Have you seen my language?*	moma.org	Eating	Art
Bread	Food recipes online	thespruceeats.com/	Eating	Not-Art
Eggs	Matt Keegan (2016), *Have you seen my language?*	moma.org	Eating	Art
Eggs	Food recipes online	taste.com.au/	Eating	Not-Art
Fork	Matt Keegan (2016), *Have you seen my language?*	moma.org	Eating	Art
Fork	Free images online	pixnio.com	Eating	Not-Art
Candy	Eyal Burstein, Michele Gauler, Beta Tank (2007), *Eye Candy from the Sensory Plasticity project (Models)*	moma.org	Eating	Art
Candy	Online selling ad	lorentanuts.com	Eating	Not-Art
Clock	Ruth Ewan (2011), *We could have been anything*	tate.org.uk	Observing	Art

	that we wanted to be (red version)			
Clock	Online selling ad	amazon.co.uk	Observing	Not-Art
Painting	Stephen Shore (1972), *Farmington, New Mexico*	metmuseum.org	Observing	Art
Painting	Online selling ad	avito.ru	Observing	Not-Art
Plant	Stephen Shore (1973), *Palm Beach, Florida*	tate.org.uk	Observing	Art
Plant	Online selling add	oxy-plants.com	Observing	Not-Art
Statue	Stephen Shore (1972) *Philadelphia, Pennsylvania*	tate.org.uk	Observing	Art
Statue	Online selling ad	avito.ru	Observing	Not-Art
Heart	Robert Gober (2014-2015), *Heart in a box*	whitney.org	Observing	Art
Heart	Online selling ad	avito.ru	Observing	Not-Art
Flowers	Urs Fischer (2011), *Necrophonia*	ursfischer.com	Observing	Art
Flowers	Online selling ad	roseandgrey.co.uk	Observing	Not-Art
Plant	Thomas Ruff (1979), *Interieur*	centrepompidou.fr	Observing	Art
Plant	Gardening website	balconygardenweb.com	Observing	Not-Art
Book	R.B. Kitaj (1972), *Men and Books*	tate.org.uk	Reading	Art
Book	Online selling ad	lanntair.com	Reading	Not-Art
Soap	Robert Watts (1966), *Flux Lux Soap*	moma.org	Showering	Art
Soap	Online selling ad	thehempstore.co.uk	Showering	Not-Art
Toothbrush	Robert Watts (1964), *Chromed Toothbrush*	moma.org	Showering	Art
Toothbrush	Online selling ad	genhealthcare.co.uk	Showering	Not-Art
Liquid soap	Matt Keegan (2016), *Have you seen my language?*	moma.org	Showering	Art
Liquid soap	Recipe website	thethingswellmake.com	Showering	Not-Art
Bed	Urs Fischer (2000), *Bed*	ursfischer.com	Sleeping	Art
Bed	Online property ad	rightmove.co.uk	Sleeping	Not-Art
Pyjama	Stephen Shore (1972), *New York, New York*	metmuseum.org	Sleeping	Art
Pyjama	Online selling ad	avito.ru	Sleeping	Not-Art
Shoes	Sherrie Levine (1992), *2 Shoes*	tate.org.uk	Walking	Art
Shoes	Online selling ad	chikoshoes.com	Walking	Not-Art
Boots	Pope.L (1991), *Tompkins Square Crawl*	moma.org	Walking	Art
Boots	Online selling ad	avito.ru	Walking	Not-Art
Trainers	Zoe Leonard (2007), *Analogue*	moma.org	Walking	Art
Trainers	Online selling ad	ebay.co.uk	Walking	Not-Art
TV	Mark Dickenson (1995), *Untitled (Clothes)*	tate.org.uk	Watching media	Art
TV	Online selling ad	amazon.co.uk	Watching media	Not-Art
Computer Mouse	Steven T. Kaneko (1992), *Mouse Computer Pointing Device*	moma.org	Working	Art
Computer Mouse	Online news outlet	telegraph.co.uk	Working	Not-Art

Computer	Jonathan Ive (2001), *Apple Industrial Design GroupiMac G4 Desktop Computer*	moma.org	Working	Art
Computer	Wallpaper website	wallpaperflare.com	Working	Not-Art
Keyboard	Apple, Inc., Stephen Peart, Raymond Riley, David Shen (1993), *Adjustable Keyboard*	moma.org	Working	Art
Keyboard	Online selling ad	ebay.co.uk	Working	Not-Art

Table S1. List of images, sources and activities

	Embodiment Score (log)		
Predictors	Estimates	CI	*p*
(Intercept)	0.31	0.26 – 0.36	**<0.001**
Condition [Commercial]	-0.04	-0.10 – 0.03	0.272
Condition [Museum]	-0.02	-0.08 – 0.04	0.570
Image Type [Art]	0.07	0.02 – 0.13	**0.009**
Body Part [Belly]	-0.32	-0.37 – -0.26	**<0.001**
Body Part [Chest]	-0.14	-0.20 – -0.09	**<0.001**
Body Part [Hands]	-0.12	-0.18 – -0.07	**<0.001**
Body Part [Legs]	-0.53	-0.59 – -0.48	**<0.001**
Condition [Commercial] × Image Type [Art]	0.00	-0.07 – 0.08	0.913
Condition [Museum] × Image Type [Art]	0.06	-0.02 – 0.13	0.157
Condition [Commercial] × Body Part [Belly]	0.01	-0.07 – 0.08	0.880
Condition [Museum] × Body Part [Belly]	0.03	-0.05 – 0.11	0.425
Condition [Commercial] × Body Part [Chest]	-0.01	-0.09 – 0.06	0.778
Condition [Museum] × Body Part [Chest]	-0.00	-0.08 – 0.08	0.980
Condition [Commercial] × Body Part [Hands]	0.04	-0.04 – 0.11	0.323
Condition [Museum] × Body Part [Hands]	0.05	-0.03 – 0.13	0.191
Condition [Commercial] × Body Part [Legs]	0.11	0.04 – 0.19	**0.004**
Condition [Museum] × Body Part [Legs]	0.09	0.02 – 0.17	**0.015**
Image Type [Art] × Body Part [Belly]	0.02	-0.06 – 0.10	0.598
Image Type [Art] × Body Part [Chest]	-0.15	-0.23 – -0.07	**<0.001**
Image Type [Art] × Body Part [Hands]	-0.11	-0.19 – -0.03	**0.004**
Image Type [Art] × Body Part [Legs]	-0.11	-0.19 – -0.03	**0.005**
(Condition [Commercial] × Image Type [Art]) × Body Part [Belly]	0.04	-0.07 – 0.14	0.501
(Condition [Museum] × Image Type [Art]) × Body Part [Belly]	-0.03	-0.13 – 0.08	0.638

(Condition [Commercial] × Image Type [Art]) × Body Part [Chest]	0.03	-0.08 – 0.13	0.634
(Condition [Museum] × Image Type [Art]) × Body Part [Chest]	-0.01	-0.12 – 0.10	0.859
(Condition [Commercial] × Image Type [Art]) × Body Part [Hands]	-0.01	-0.12 – 0.10	0.880
(Condition [Museum] × Image Type [Art]) × Body Part [Hands]	-0.10	-0.21 – 0.01	0.069
(Condition [Commercial] × Image Type [Art]) × Body Part [Legs]	-0.07	-0.18 – 0.04	0.187
(Condition [Museum] × Image Type [Art]) × Body Part [Legs]	-0.07	-0.18 – 0.04	0.209

Random Effects

σ^2	0.56
$\tau_{00\ pid}$	0.01
$\tau_{00\ img\_num}$	0.00
ICC	0.02
N_{img_num}	34
N_{pid}	265
Observations	45050
Marginal R^2 / Condition al R^2	0.055 / 0.077

Table S2. The Table above reports the results of the Mixed-Effects Model Assessing the Effect of Condition (Mixed *vs* Commercial *vs* Museum), Image Type (Art *vs* Not-Art), and Body Part (Head *vs* Chest *vs* Abdomen *vs* Upper Limbs *vs* Lower Limbs) on Embodiment Score. The reference level for Condition is Mixed, for Image Type is Not-Art, and for Body Part is Head. The bolded p-values are those which are statistically significant.

Objects	Activity	Frequency
apple, bacon, banana, biscuit, bowl, bread, breakfast, broccoli, butter, cabbage, carrots, cereal, cheese, chicken, chips, chocolate, coffee, crisps, cutlery, curry, eggs, fire, fish, flame, fork, form, fruit, ham, honey, kebab, kimchi, knife, lettuce, mach, meatballs, mug, mustard, oven, pan, pasta, peanuts, plate, porridge, potato, rice, sandwich, sausages, scampi, soup, spices, spoon, stir fry, tea, toast, tomato sauce, tomatoes, vegetables, yoghurt	Eating	36
bag, ball, bus, boots, coat, dog, fitness tracker, fresh, gloves, hat, hood, hoodie, jacket, jeans, keys, kindle, lead, outside, park, path, poo bags, purse, rain, scarf, school, sea front, shoes, shop, socks, sun, toddler, trainers, t-shirt, umbrella, work	Walking	27
bath, bowl, cloth, conditioner, deodorant, flannel, hot water, makeup, mouthwash, shampoo, shower, shower gel, shower head, soap, toothbrush, toothpaste, towel, washing liquid, water, shaving razor	Showering/ washing/ bathing	18
alarm, bed, blanket, dressing gown, duvet, hot water bottle, mattress, pillows, PJ, safety, tablet	Sleeping	14
bricks, camera, chair, computer, desk, diary, dogs, earning online, headset, internet, keyboard, laptop, meetings, monitor, mouse, notebook, papers, printer, setup, smartphone, speakers, tripod	Working	13
cardigan, clothes, coat, deodorant, gloves, handbag, hat, jeans, jumper, leggings, PJ, scarf, shoes, slippers, socks, sweater, trousers, t-shirt, vest, wardrobe	Dressing	11
call the midwife, Disney, headphones, ITV, music, Netflix, podcasts, remote control, screen, smartphone, social media, sofa, speakers, spotify, twitch streams, TV	Watching/ listening media	11
beer, coffee, coke, diluted juice, glass, mug, orange juice, pepsi, spoon, sugar, tea, water, wine	Drinking	10
biscuit, book, bookmark, cake , chair, coffee, e-book, kindle, lamp, light, magazine, mail, online news, tablet, words	Reading	9
bins, bleach, brush, cloth, clothes, duster, dusting, hoover, hoovering, kitchen, laundry liquid, loths, mop, polish, pressure washer, scourer, sloths, soap bar, softener, spray, steam, towels, vacuum cleaner, washing dishes, washing liquid, water, water sponge, broom	Cleaning	9
car, car keys, coat, diesel, my Fiesta, music, road	Driving	9
colander, crockery, fork, fresh, frying, frying pan, heat, hob, homemade, knife, microwave, olive oil, oven, pan, pepper, salt, saucepan, smell, spoon, tray, utensils, vegetables, saucepan	Cooking	8
bench, earbuds, football, headphones, jump rope, leggings, music, phone, running shoes, shoes, shorts, towel, trainers, treadmill, t-shirt, water bottle, wind, weights	Exercise	7

friends, mom, laptop, telephone, mobile phone	Talking	7
chair, cushions, throws, radio, book, candy crush, TV, laptop	Sitting	4
bank card, bag, money, list	Shopping	3
bus, ticket, metro, bike	Commuting	3
cloth, machine, thread, needle, scissors	Sewing	2
grand son, laughter, love, breakfast, cereal, bowls, spoons, toothbrush, uniform, shoes, backpack	Childcare	2
lawn mower	Gardening	2
fifa, console	Gaming	2
flowers	Watering	1
mirror, light, brush, pink	Applying makeup	1
cigarette	Smoking	1
excel	Budgeting	1

Table S3. The table above lists all the objects (in the Objects column) with their associated activities (in the Activity column) and the number of times each activity was mentioned in the pilot study (in the Frequency column). The top ten most frequently mentioned actions are highlighted in grey.

Condition	Instructions
**Museum**	‘In this study we investigate how people perceive contemporary art. You will see a set of images made by professional artists. They were exhibited in contemporary art galleries all over the world, including Tate Modern in London and the Museum of Modern Art in New York. All artworks you will see represent the found art. Found art is art created from everyday life objects or products that are not normally considered materials from which art is made, often because they already have a non-art function. By choosing the object and repositioning or joining, signing it, the found object became art.’
**Commercial**	‘In this study we investigate product perception. You will see a set of images made for sale online. These images represent both new and used products. Some images you will see are made by companies which sell brand new products online, including furniture, food, medical items, clothing and others. Some products were photographed by people who wish to sell used items online on a website like ebay.co.uk’
**Mixed**	‘In this study we investigate image perception. You will see a set of images. Some of them were made by professional artists or by people for sale online. Images made by the artists were exhibited in contemporary art galleries all over the world, including Tate Modern in London and the Museum of Modern Art in New York. Images made by people represent both new and used products for sale.’

Table S3. The table above reports the specific instructions provided to participants in each condition (museum, commercial, and mixed) to induce a distinct cognitive stance.

Supplementary Figures

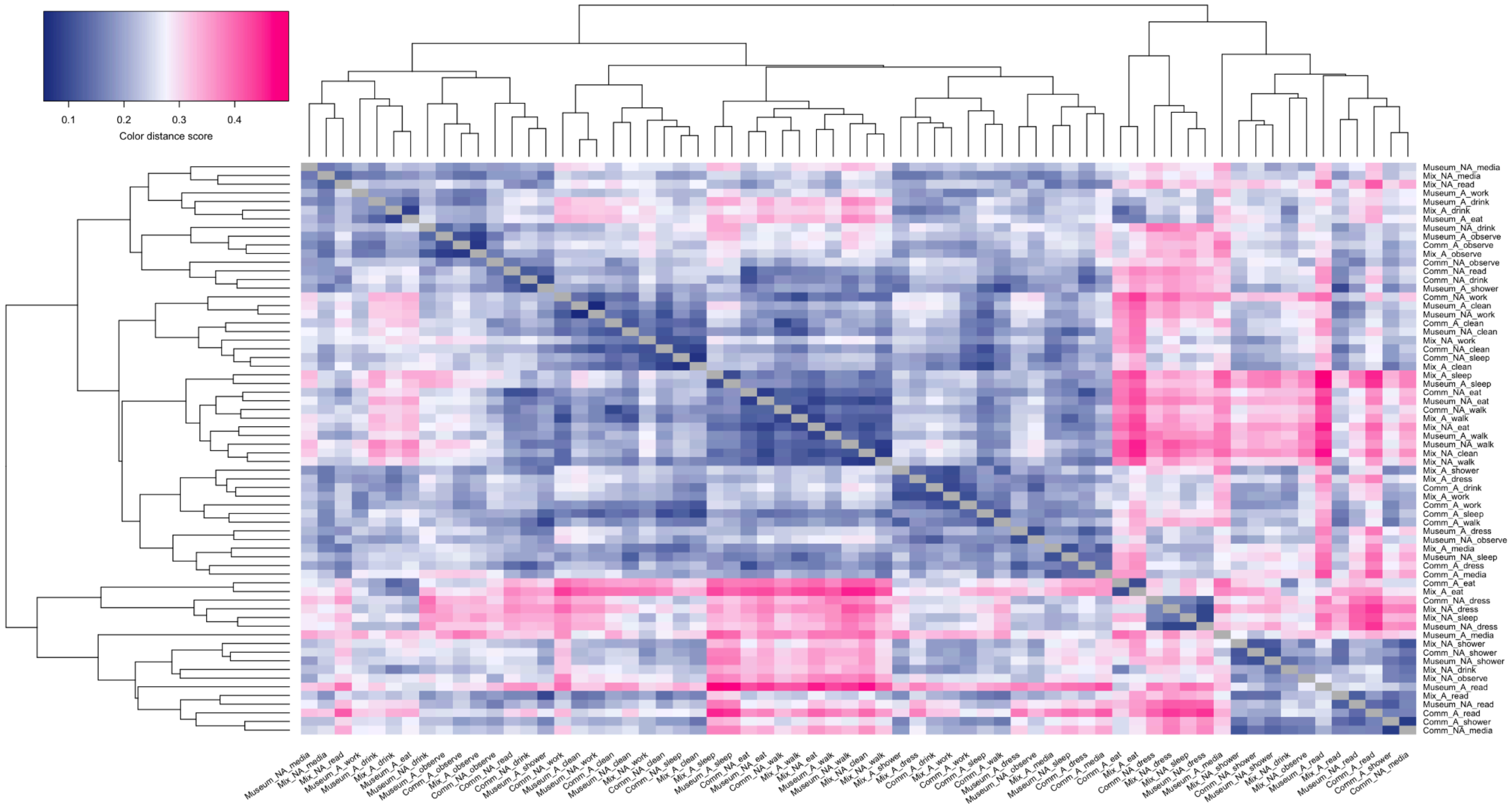

Figure S1. Distance matrix of colour (dis)similarity for BSMs across all levels of Conditions, Image Type, and Activities.
Note. Comm = Commercial; Museum = Museum; Mix = Mixed; A = Art; NA = Not-Art;
eat = eating; walk = walking; sleep = sleeping; work = working; shower = showering; dress = dressing; media = watching media, drink = drinking; read = reading; clean = cleaning; dress = dressing; observe = observing

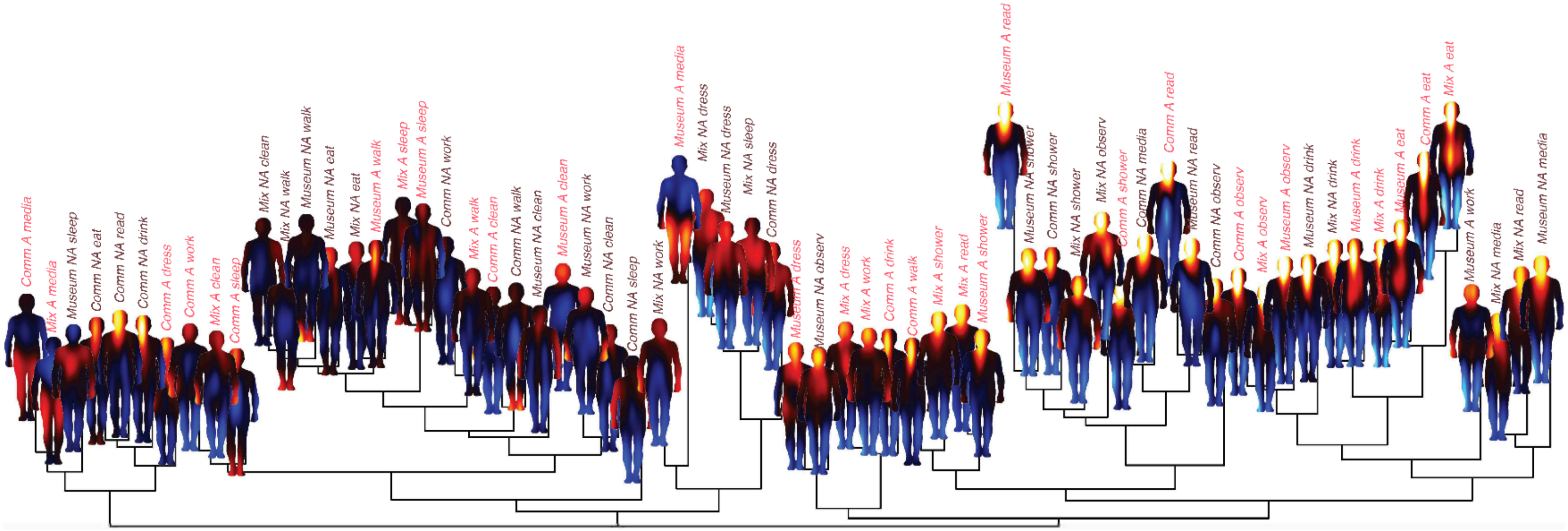

Figure S2. Clustering Tree of BSMs across Conditions, Image Type, and Activities
Note. Comm = Commercial; Museum = Museum; Mix = Mixed; A = Art (light red); NA = Not-Art (dark red);
eat = eating; walk = walking; sleep = sleeping; work = working; shower = showering; dress = dressing; media = watching media;
drink = drinking; read = reading; clean = cleaning; dress = dressing; observe = observing
